# Supplementary material for: Structural changes in noble metal nanoparticles during CO oxidation and their impact on catalyst activity
Source: Nat Commun. 2020 May 1;11:2133. doi: 10.1038/s41467-020-16027-9 (PMC7195460; doi:10.1038/s41467-020-16027-9)
Supplement: Supplementary file 1 — Supplementary Information [file 41467_2020_16027_MOESM1_ESM.pdf]

Supplementary Information for

Structural Changes in Noble Metal Nanoparticles during CO Oxidation and Their Impact on Catalyst Activity

Chee *et al.*

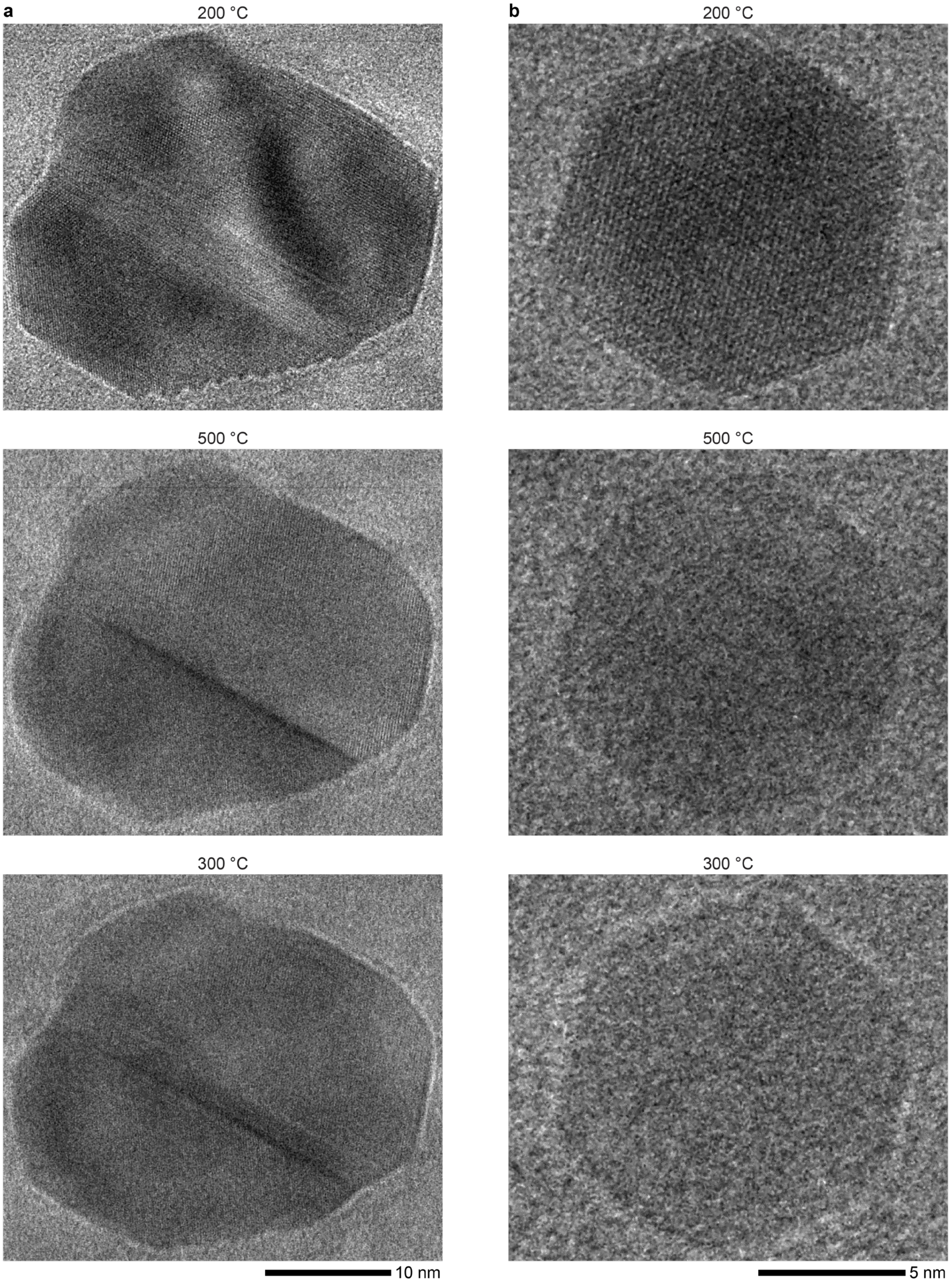

Supplementary Figure 1. Enlarged images of the two Pd NPs shown in the main text. (**a**) Figure 1 and (**b**) Figure 4 for temperatures of 200, 500, and 300 °C with $\frac{P_{CO}}{P_{O_2}} = 0.5$.

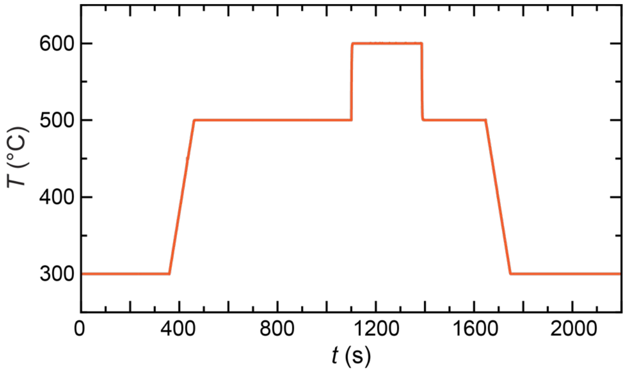

Supplementary Figure 2. Heating profile for the experiment described in the main text Figure 1 and 2.

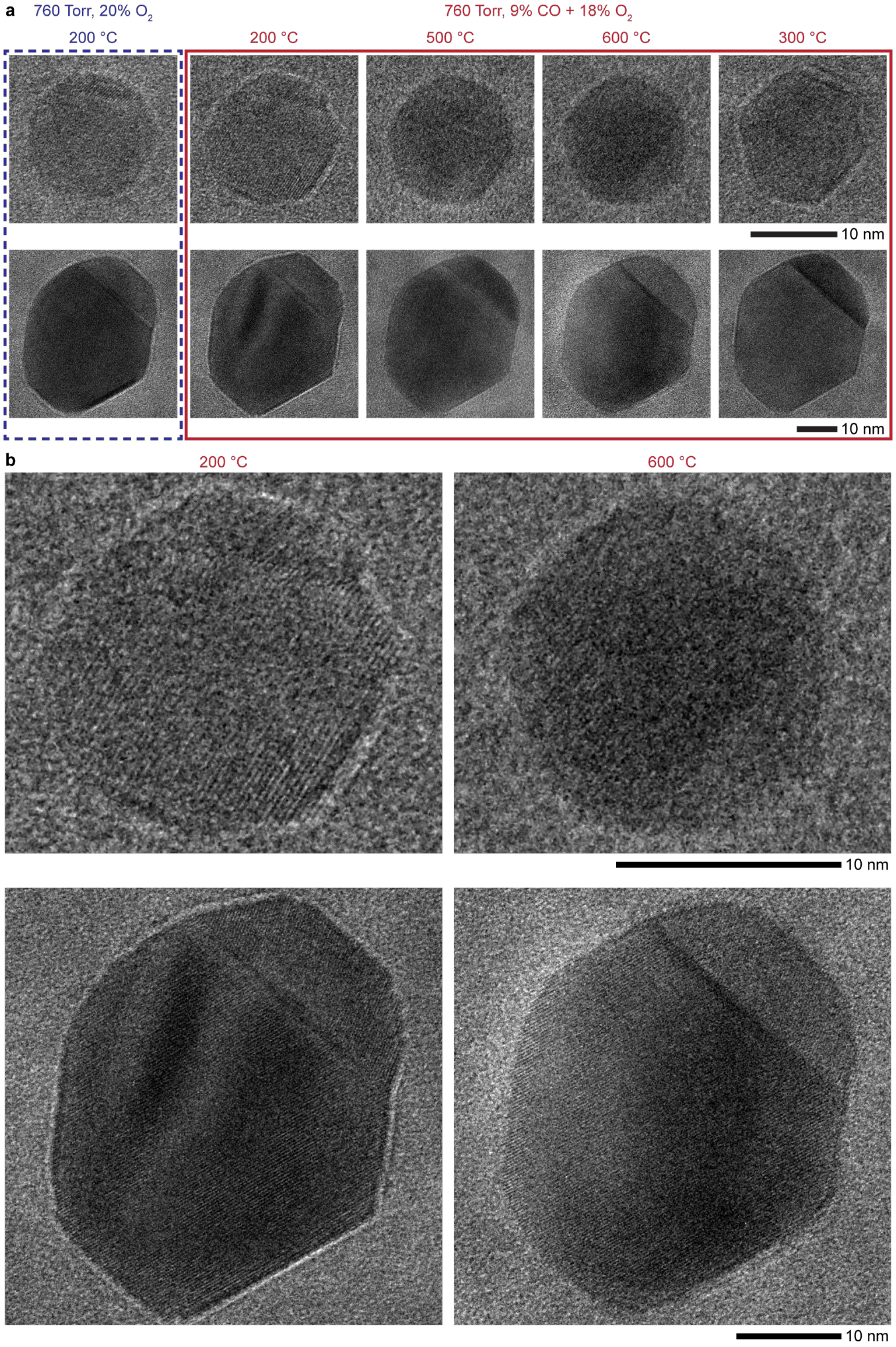

Supplementary Figure 3. Images of two other Pd NPs acquired with $\frac{P_{CO}}{P_{O_2}} = 0.5$ and at different temperatures. (**a**) Image sequence of the two NPs. The first two pairs of images in the red box show that the NPs formed flat facets and sawtooth steps when CO was introduced at 200 °C. The NPs became more rounded after heating to 500 and 600 °C, which also corresponded with the NPs becoming active. The flat facets and sharp corners re-formed when the temperature was lowered back to 300 °C. (**b**) Enlarged images of the two NPs at 200 and 600 °C.

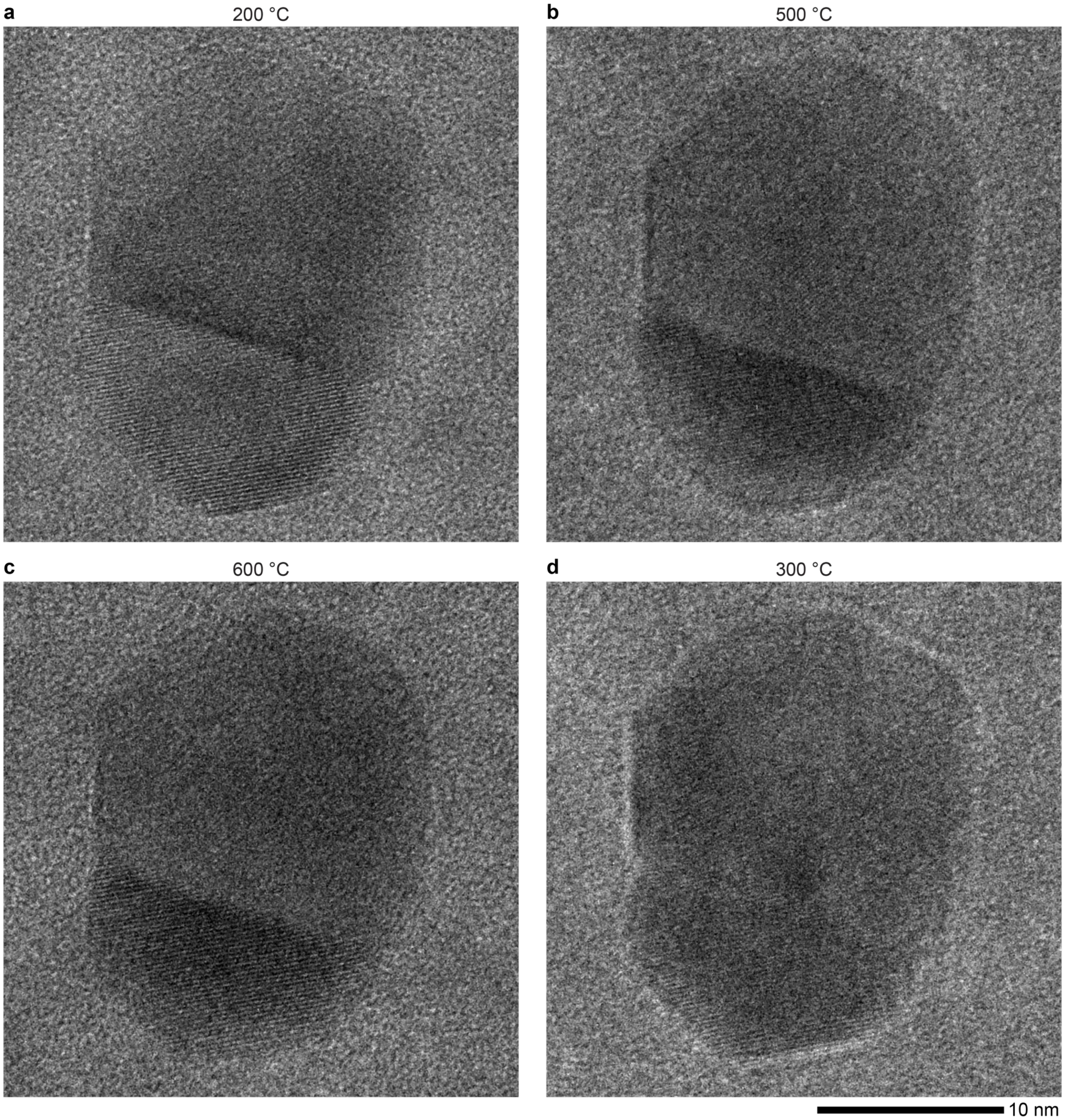

Supplementary Figure 4. Image sequence showing enlarged images of another Pd NP acquired at different temperatures with $\frac{P_{CO}}{P_{O_2}} = 0.5$. The NP at (**a**) 200 °C, (**b**) 500 °C, (**c**) 600 °C, and (**d**) 300 °C. The heating sequence was the same as Supplementary Figure 3.

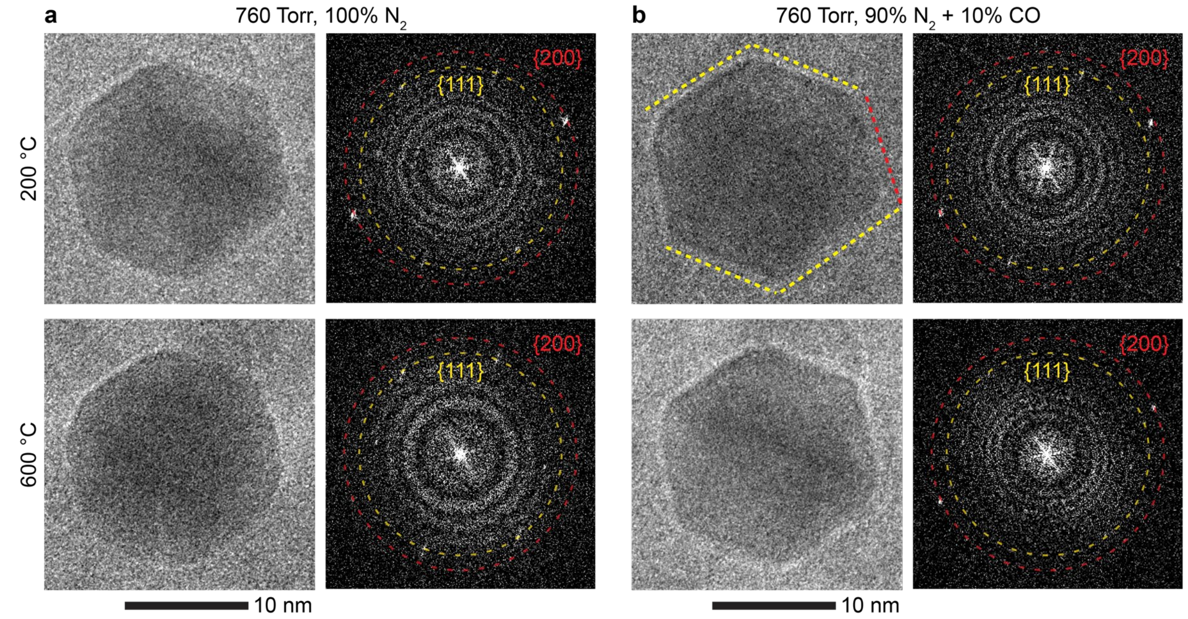

Supplementary Figure 5. Structure of a Pd NP in N_2 and N_2 + CO gas environments. Images of a Pd NP and their corresponding fast Fourier transforms (FFT) acquired under (**a**) 760 Torr pure N_2 and (**b**) 760 Torr 90% N_2 + 10% CO atmosphere. In the N_2 environment, the NP becomes rounded when heated to 600 °C, presumably due to the desorption of adsorbed CO molecules. With CO added into the gas mixture, the low index facets appear to be dynamically stabilized up to 600 °C. Yellow and red dashed lines indicate the {111} and {100} facets, respectively.

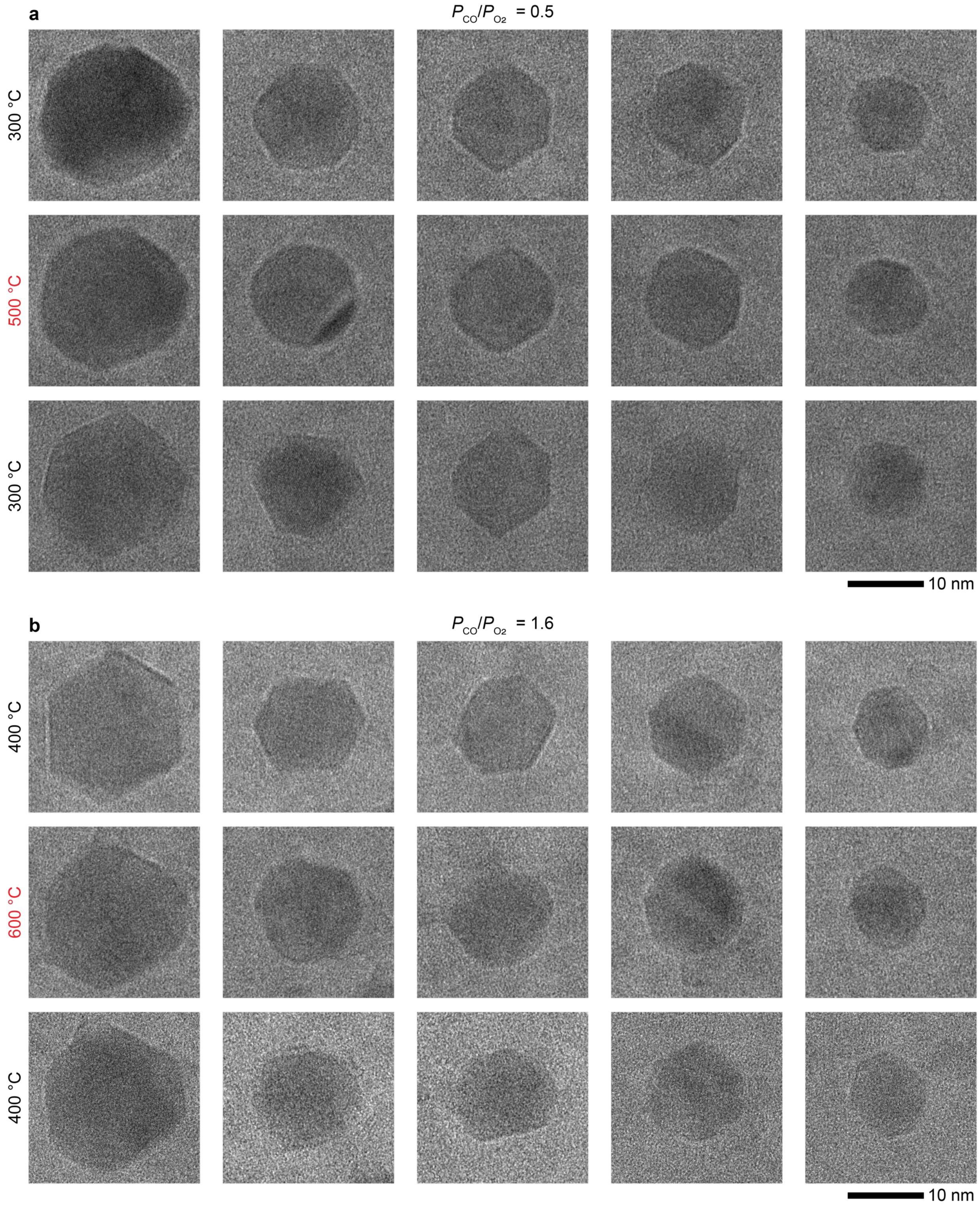

Supplementary Figure 6. Five Pd NPs imaged under different CO to O_2 ratios and at different temperatures. The NPs are inactive at 300 – 400 °C for both conditions and are at (**a**) 500 °C for $\frac{P_{CO}}{P_{O_2}} = 0.5$ and (**b**) 600 °C for $\frac{P_{CO}}{P_{O_2}} = 1.6$.

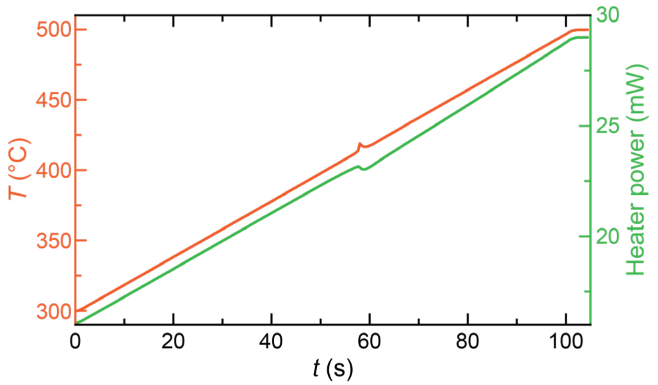

Supplementary Figure 7. Measured temperature and heating power profiles during the temperature ramp from 300 to 500 °C for the experiment described in Supplementary Figure 6a at $\frac{P_{CO}}{P_{O_2}} = 0.5$. The ignition temperature for this experiment was measured to be ~ 420 °C.

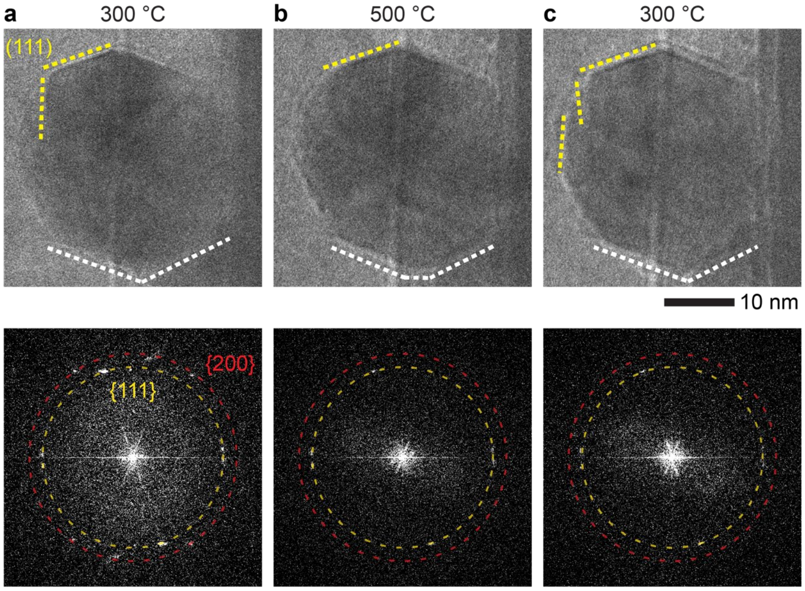

Supplementary Figure 8. Additional Pd NP imaged under $\frac{P_{CO}}{P_{O_2}} = 1.6$. Images of the Pd NP at (**a**) 300 °C, (**b**) 500 °C, and back at (**c**) 300 °C under conditions similar to that described in Figure 3 and Supplementary Figure 6b and their corresponding FFT. In this case, the NP was found on the vertical edge of the SiN_x window, and so, the NP could be seen from a different perspective. The yellow dashed lines denote {111} facets, whereas the white dashed lines denote where the rounding of one NP corner at 500 °C. The top of the NP also changes its structure at the different temperatures in a manner consistent with the behaviors described in the main text.

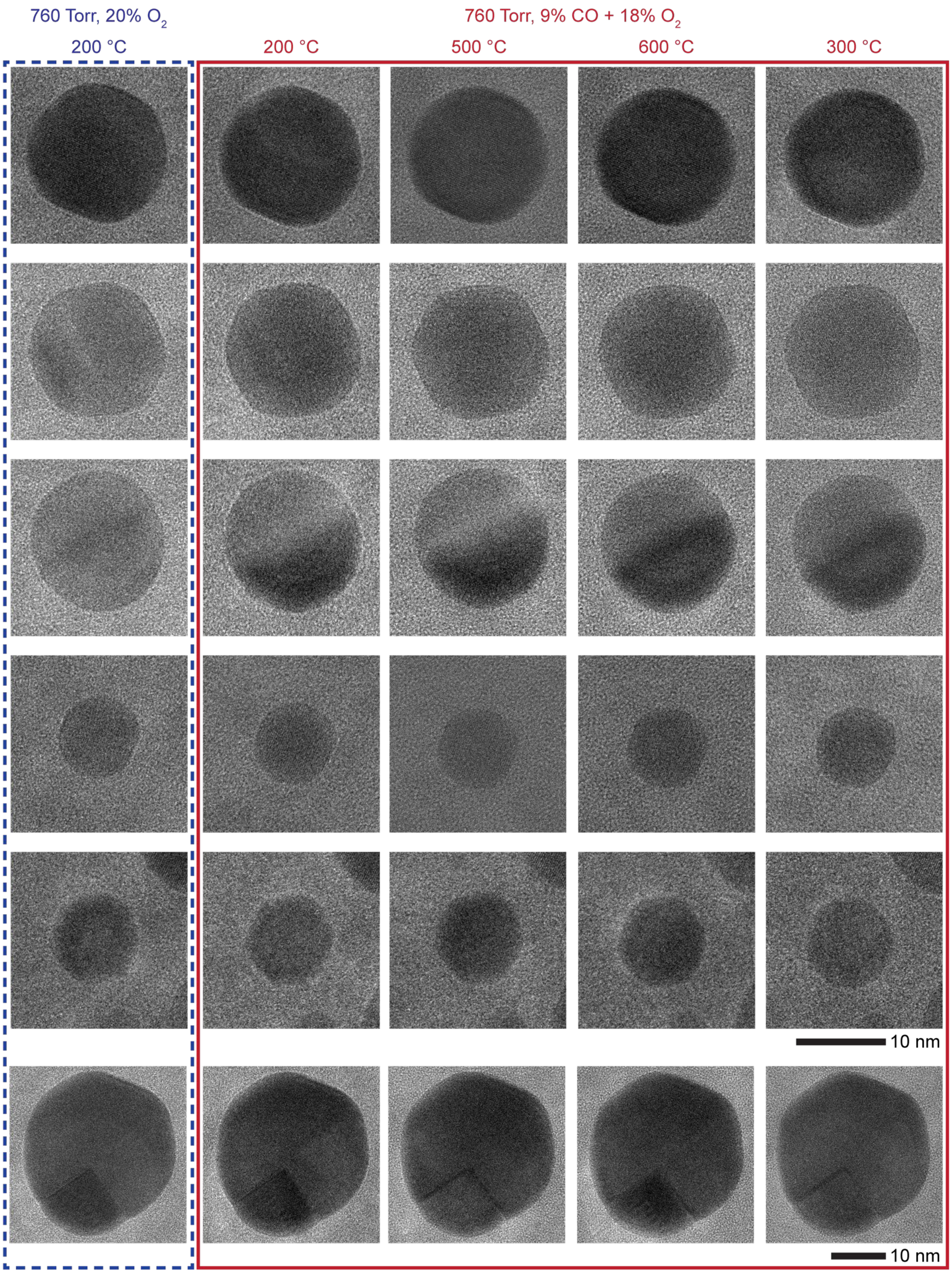

Supplementary Figure 9. Six other Pt NPs imaged under the same conditions as described in Figure 4. These images highlight the subtle re-faceting that was seen in the Pt NPs during CO oxidation. At 500 and 600 °C, there appeared to be faceting in parts of the NPs, but the difference between these more active structures and the less active ones at lower temperatures was not as obvious as that seen in Pd NPs.

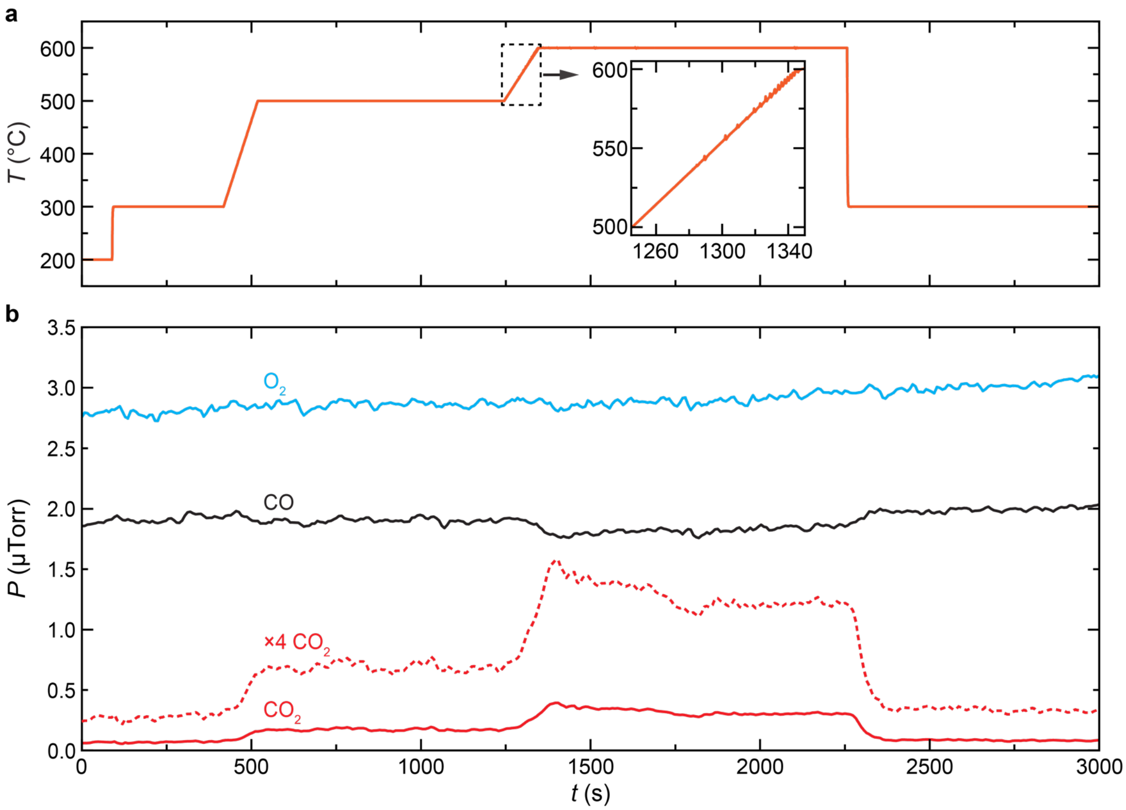

Supplementary Figure 10. Measured temperature profile and mass spectrometry results from the CO oxidation experiment involving Pt NPs described in Supplementary Figure 9. (**a**) Temperature profile from the experiment. The insert in the profile highlights a series of peaks seen in the measured temperature during the ramp from 500 to 600 °C (black dashed box). We speculate that the series of peaks seen during the temperature ramp was not due to ignition but is related to the oscillatory behavior reported by Vendelbo *et al*.[1] The temperature range over which the series of peaks appeared is consistent with the temperature range reported by the authors for the observed CO_2 conversion oscillations. (**b**) Gas composition changes during the experiment. The red dash line indicates the CO_2 profile multiplied by 4× to accentuate the activity changes at different temperatures. The amount of CO_2 detected gradually increased after the temperature was raised to 300 °C. The subsequent temperature ramps to 500 and 600 °C resulted in increasing amounts of CO_2 detected, which decreased when the temperature was reduced to 300 °C.

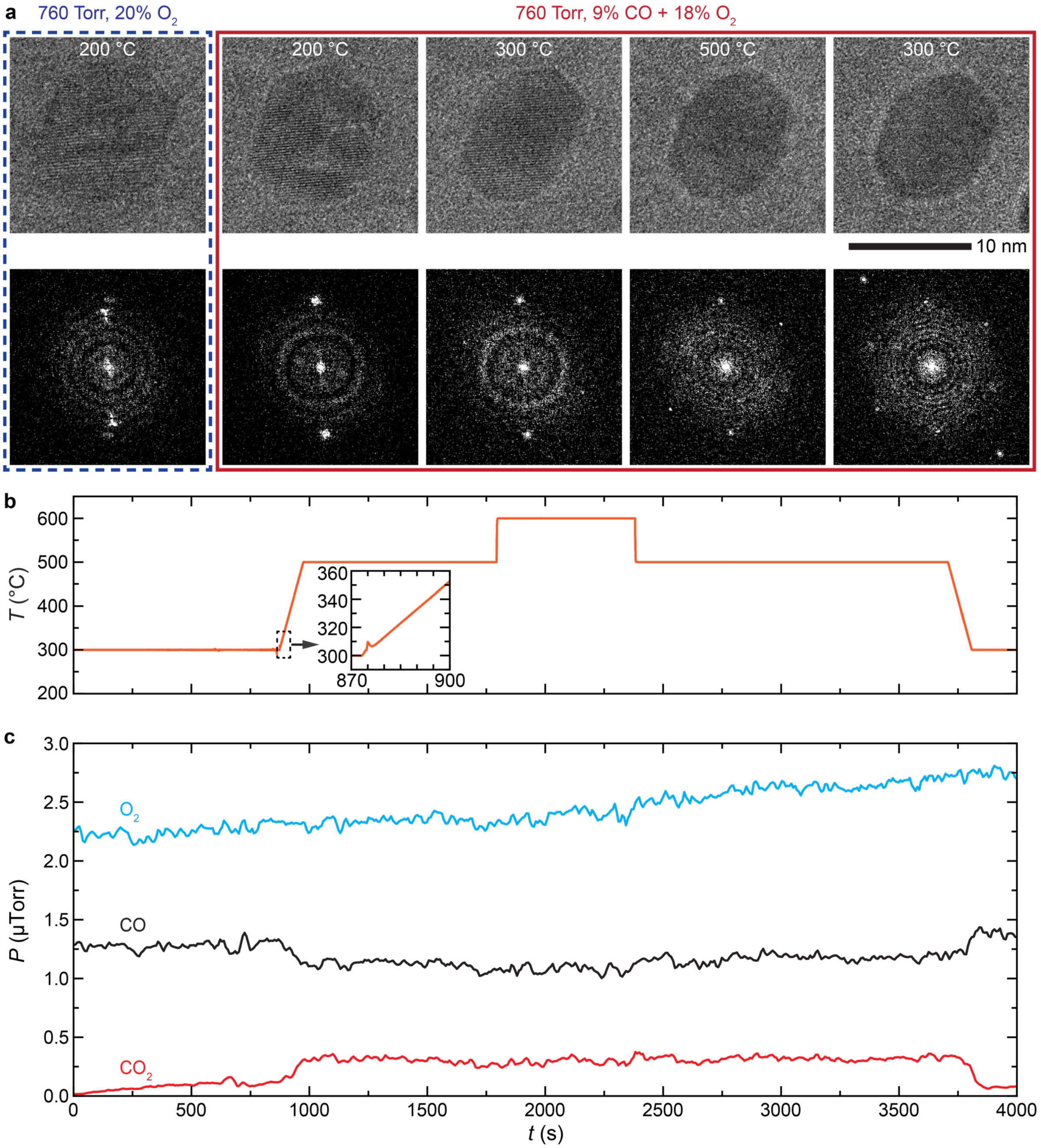

Supplementary Figure 11. *Operando* TEM of Rh NPs. (**a**) Image sequence of the Rh NP from the main text Figure 4. The FFT extracted from the image sequence indicates that the Rh NP changed from an oxide phase to a metallic phase over the course of the experiment. (**b**) Measured temperature profile and (**c**) mass spectrometry results from this experiment. The measured temperature plot shows a small spike (enlarged in inset) at just above 300 °C during the ramp from 300 to 500 °C (black dashed box), which indicates a catalytic ignition. The mass spectrometry results show that CO_2 production gradually increased when the samples were held at 300 °C. There was a further increase in CO_2 after the temperature ramp to 500 °C, but there was no increase when we raised the temperature to 600 °C. The production dropped when the temperature was reduced back to 300 °C, but no re-oxidation of the NP was observed.

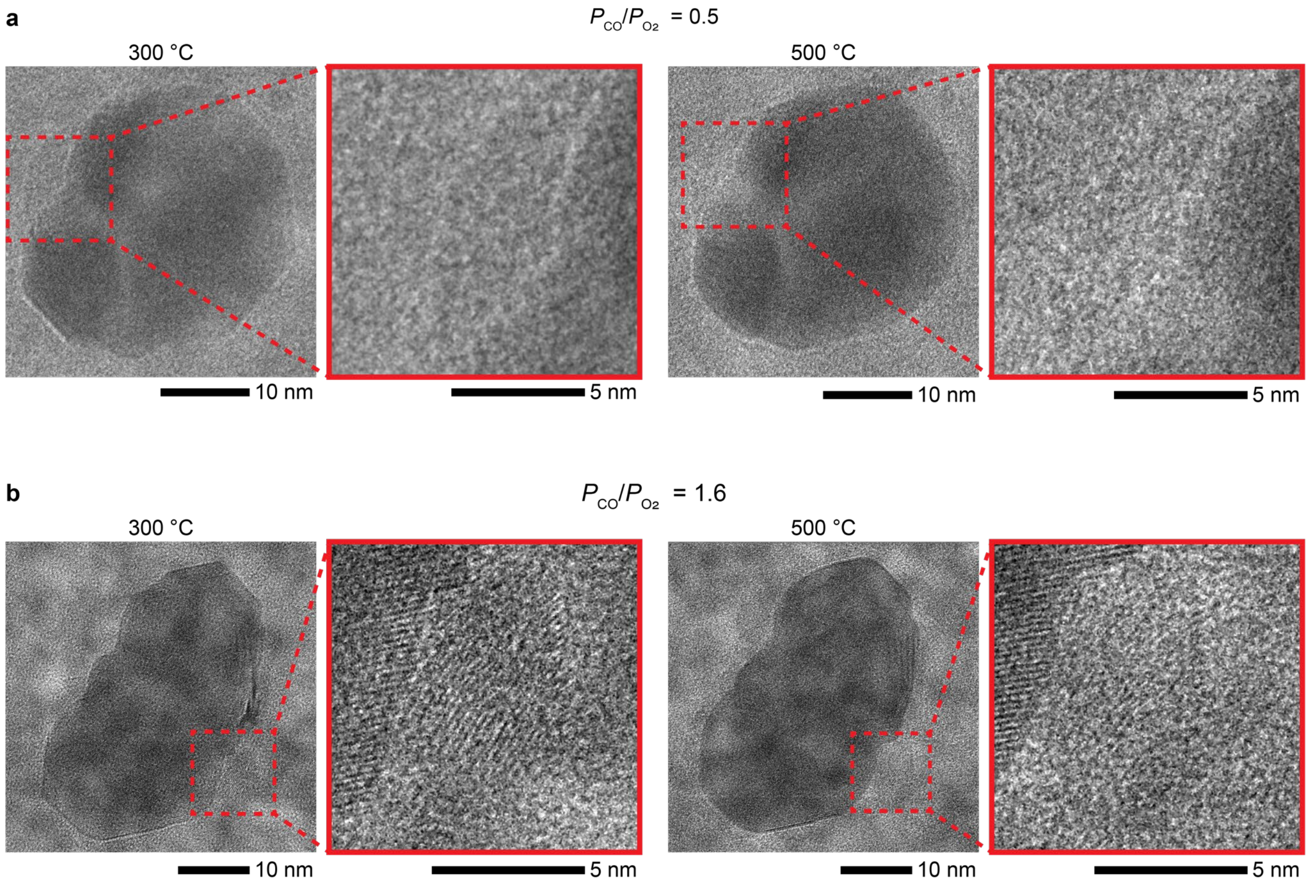

Supplementary Figure 12. Images showing possible bulk PdO on larger Pd NPs at for two temperatures of 300 and 500 °C. (**a**) Images acquired from an experiment with $\frac{P_{CO}}{P_{O_2}} = 0.5$. (**b**) Images acquired from an experiment with $\frac{P_{CO}}{P_{O_2}} = 1.6$. The areas indicated by the red dashed boxes are enlarged. The lattice fringes in the enlarged images for (**b**) show lattice fringes of ~ 0.204 nm.

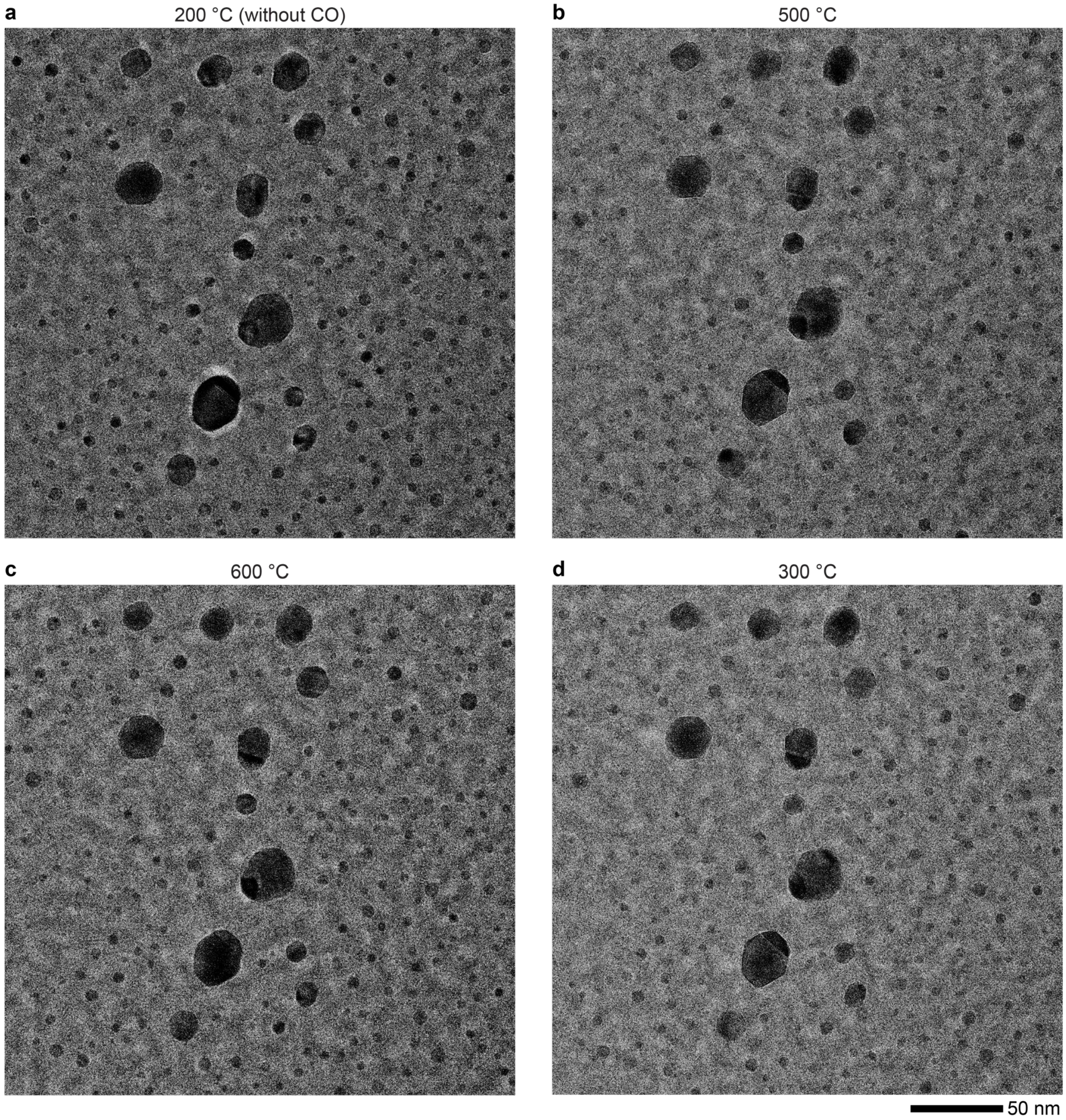

Supplementary Figure 13. Low magnification images of Pd NPs during an experiment. (**a**) At the start of the experiment where the conditions were 1 bar of 80% He and 20% O_2 at 200 °C and (**b-d**) during the experiment with 1 bar 73% He, 18% O_2, and 9% CO at 500, 600, and 300 °C, respectively. These images are representative of the NP loading observed during our experiments and show that the NPs are well-separated from each other with no NP coalescence occurring over the course of the experiment.

Supplementary Note

DFT Calculations

All calculations were carried out using density functional theory (DFT) with the BEEF-vdW functional implemented in VASP 5.4.4.[2–5] The projector-augmented wave (PAW)[6] method was used to treat the core electrons. Plane-wave basis sets with a 400-eV cutoff energy were used to model the 10 valence electrons for Pd, 10 for Pt, 4 for C, and 6 for O. A characteristic parameter with a width of 0.2 eV for the first–order Methfessel–Paxton smearing technique was used, and the total energies were extrapolated to zero width. We have set the SCF convergence criterion to 10^{-5} eV, and the convergence threshold criterion to 2×10^{-2} eV/Å for the energy gradient of each atom used for geometry optimizations.

The surfaces (111) and (100) of Pd or Pt were represented by a slab model of five atomic layers with a 3×3 supercell for the calculation of low coverage adsorption of CO and O. The (110) facet was modeled with seven layers and a 2×3 supercell. The corresponding facets were cut from the optimized bulk unit cells of Pd or Pt. The vacuum distance between the neighboring slabs was set to 10 Å. The atoms in the two bottom layers were fixed at the optimized bulk positions, while the rest were allowed to relax during the optimization. Slabs with a single unit cell were used to compute the CO–CO and O–O interaction energies (w_{CO} and w_{O}). Surface slabs with the dimensions of 2×2, 2×2, and 1×2 unit cells with the exposed (111), (100), and (110) facets were used to compute the CO–O interaction energy ($w_{\mathrm{CO,O}}$). In these tests, one mono-layer of CO and O were placed at alternate adsorption sites and $w_{\mathrm{CO,O}}$ was calculated according to the scheme reported previously.[7] The integration over Brillouin zone was sampled by a grid of 6×6×1, 6×6×1, and 8×6×1 using Monkhorst-Pack scheme for the large slabs in (111), (100), and (110) surface models and adjusted accordingly for reduced slab models. For the gas-phase molecules CO and O_2, a cubic unit cell with 15 ×15 × 15 $Å^3$ was calculated at the gamma point.

Fowler-Guggenheim isotherm equations

The Fowler-Guggenheim isotherm (F-G) was used to model the adsorption of CO and O_2. The F-G isotherm is a modification of the Langmuir isotherm derived from rate law expressions to account for adsorbate-adsorbate interactions. For single-component adsorption, the F-G isotherm is:

$$\frac{\theta}{1-\theta} = P\,K\,\exp\left(\frac{zw\theta}{RT}\right)$$

where R is the gas constant, T is the temperature, θ is the surface coverage, K is the adsorption equilibrium constant, P is the pressure, z is the number of neighbors of the adsorption site, and w is the adsorbate-adsorbate interaction energy.[7–9] The adsorption equilibrium constant has the form:

$$K_i = \exp\left(\frac{E_{\text{ads},i} - T\Delta S_i}{RT}\right)$$

For the adsorption of a gas mixture of CO and O_2, the F-G is extended to:

$$\frac{\theta_{\text{CO}}}{1 - \theta_{\text{CO}} - \theta_{\text{O}}} = P_{\text{CO}}\, K_{\text{CO}} \exp\left(\frac{z\left(w_{\text{CO}}\theta_{\text{CO}} + w_{\text{CO,O}}\theta_{\text{O}}\right)}{RT}\right)$$

$$\frac{\theta_O^2}{(1 - \theta_{\text{CO}} - \theta_{\text{O}})^2} = P_{\text{O}}\, K_{\text{O}} \exp\left(\frac{z\left(w_{\text{CO,O}}\theta_{\text{CO}} + w_{\text{O}}\theta_{\text{O}}\right)}{RT}\right)$$

The squared coverage terms in the adsorption isotherm of O_2 are due to the consideration of dissociative adsorption.

Modeling equilibrium structures of Pd and Pt NPs under experimental conditions

We modeled the equilibrium structures of Pd and Pt NPs with a diameter of 13 nm at conditions similar to those used in the *operando* TEM experiments: CO and O_2 partial pressures of 68 Torr (~9% of 760 Torr) and 136 Torr (~18% of 760 Torr) within a temperature range of 200 – 600 °C. We carried out the DFT calculations using the BEEF-vdW functional as described above to determine adsorption energies and adsorbate-adsorbate interaction energies following a previously proposed scheme.[7,9,10] The accurate calculation of both surface and adsorption energies is a common challenge for functionals of the generalized gradient approximation type, for which the BEEF-vdW functionals represent a good compromise.[11] Therefore, we used these latter DFT energies to compute the thermodynamic data required for the construction of Pd and Pt NPs. At the gas-solid equilibrium, the process can be modeled with the F-G isotherms to consider lateral interactions between the adsorbates, as described above. The calculated surface energies were normalized considering the experimental value of the (111) crystal planes of Pd and Pt.

In Supplementary Figure 14, we illustrate the transformation of the NP shape as the temperature increases. Here, we quantified the fraction of atomic sites exposed per facet and the fraction of metal atoms that were determined to be at the boundaries between these facets, which we define as under-

coordinated edge sites. For Pd, there is a sharp increase (> 2×) in the fraction of edge sites from 5% to 11% in a 25 °C frame starting at 275 °C (Supplementary Figure 14a). This modification occurs due to the sudden shift from a (110) faceted NP to a multi-faceted particle at higher temperatures. In contrast to Pd, there is only a gradual increase in the number of edge sites (8% to 10%) over the entire temperature range for Pt (Supplementary Figure 14b). At temperatures above 500 °C, the number of the edge sites of Pd NPs with adsorbed CO is higher in comparison to bare NPs (8%). Although we have not considered higher index facets, such as (211), we expect that they would only increase the number of edge atoms, if they are stable with CO ligands. In short, our modelling results support the experimental observations from our *operando* TEM studies where the CO + O_2 atmosphere induced sharp changes on the structure and the activity of Pd NPs and a more subtle effect on the activity of Pt NPs. These slower modifications on Pt NPs might not be easily resolved during the experiments.

The adsorption of CO molecules on metal surfaces is known to be a challenge for standard DFT calculations because of the so-called CO adsorption puzzle.[12] For example, models commonly predict adsorption at the 3-fold hollow site of a Pt (111) surface instead of adsorption at the top site as reported in experiments.[12] We also tested the improvement in the accuracy of the adsorption energies calculated with the BEEF-vdW functional. We note that the BEEF-vdW CO adsorption energies are closer to the ones measured experimentally in comparison with previous calculations for both Pd and Pt surfaces,[7,9] as shown in Supplementary Figure 15.

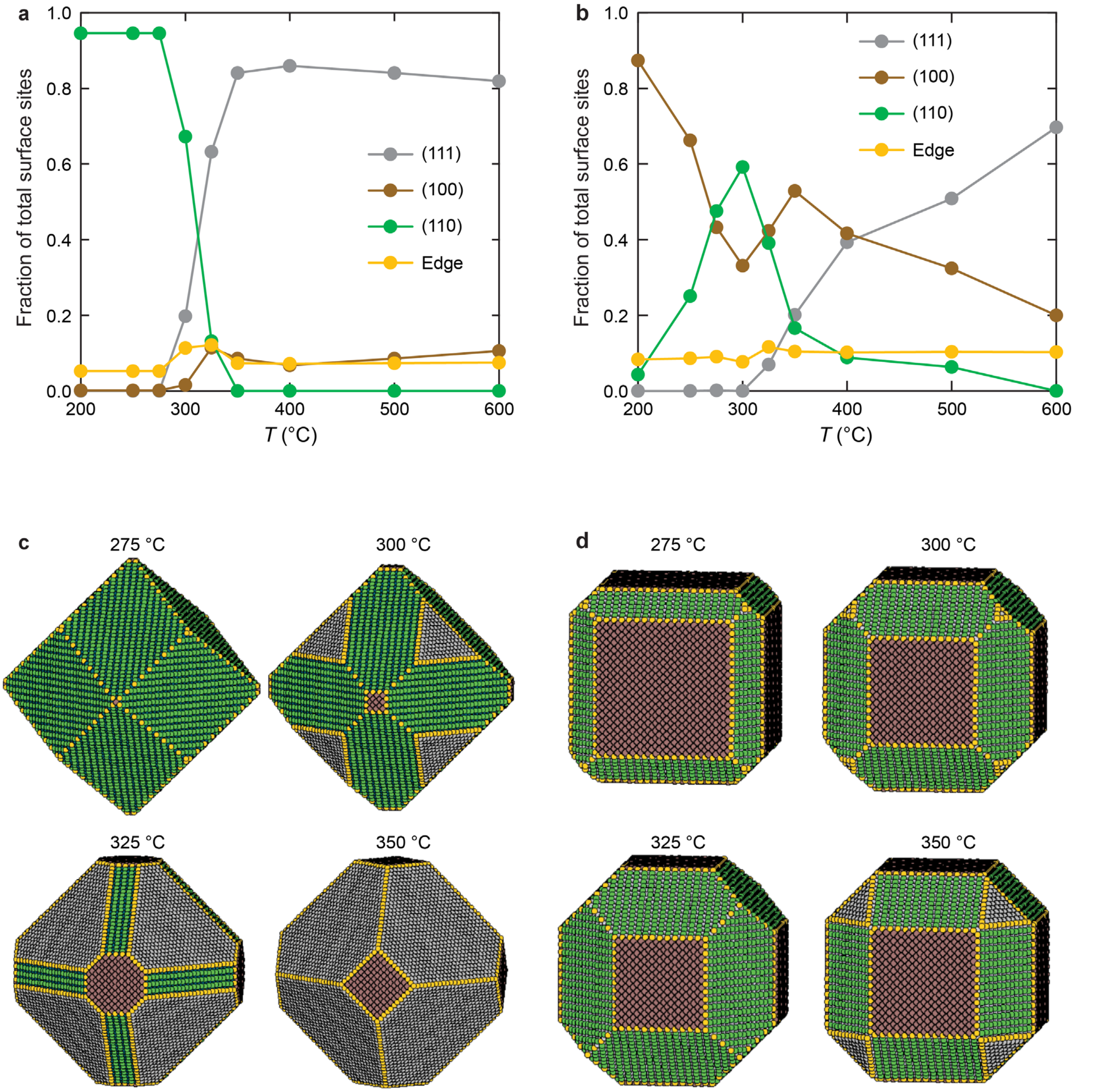

Supplementary Figure 14. Modeling the equilibrium structures Pd and Pt NPs using DFT and Wulff construction. Fraction of surface metal atoms forming (111), (100), (110) facets, and edge sites in (**a**) Pd and (**b**) Pt NPs as a function of temperature. (**c**) Equilibrium shapes of a 13 nm Pd NP under 68 Torr of CO and 136 Torr of O_2 at 275, 300, 325, and 350 °C. Color coding of the facets follows the colors used in the plots in the panel (a). The models show that the increase in the number of edge sites is due to the multi-faceted nature of the NP at higher temperatures. (**d**) Equilibrium shapes of a 13-nm Pt NP under 68 Torr of CO and 136 Torr of O_2 at 250, 300, 325, and 350 °C. Color coding of the facets follows the colors used in the plots in the panel (b).

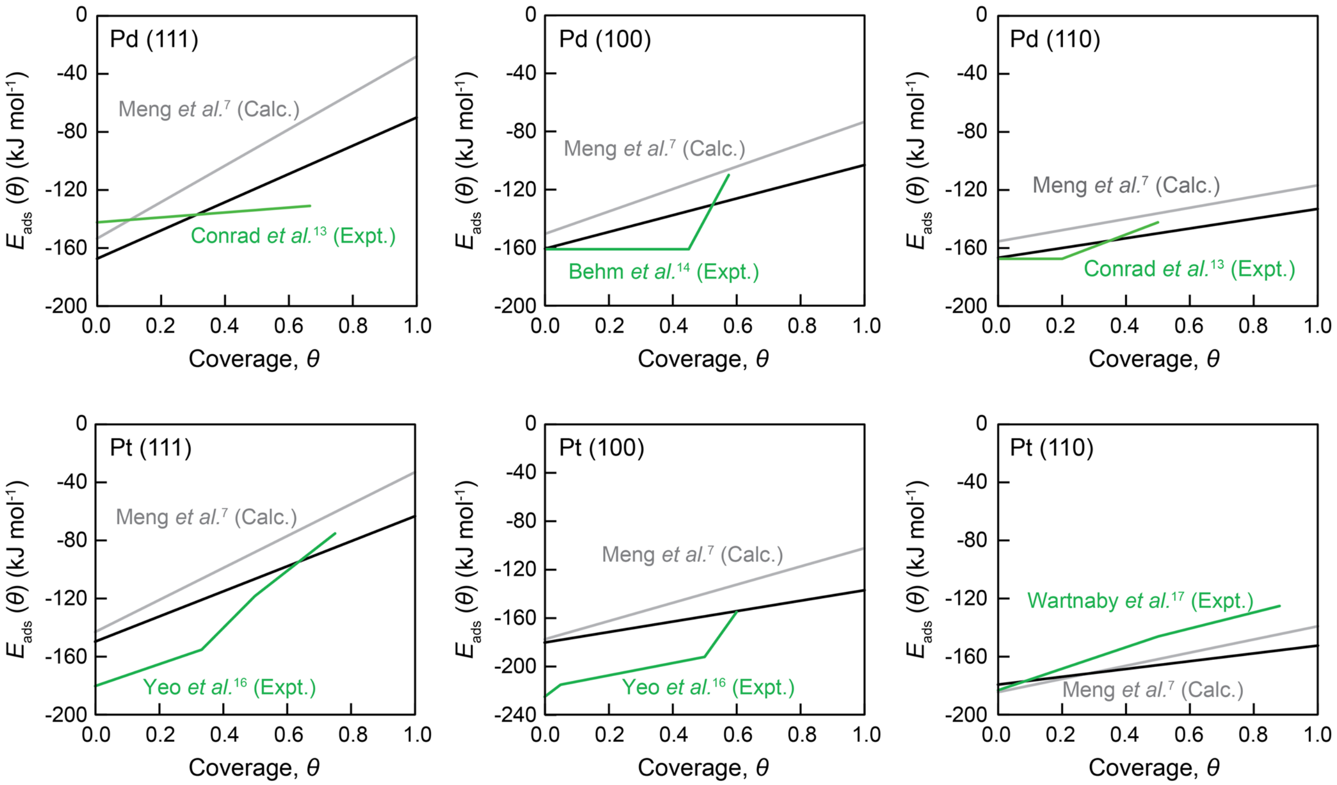

Supplementary Figure 15. A comparision of coverage-dependent adsorption energies of CO over Pd and Pt surfaces calculated in this study with previous work. Black lines show the BEEF-vdW adsorption energies of CO calculated in this study. Gray lines represent the change in CO adsorption energy with coverage from earlier models.[7] Green lines represent CO adsorption energies that were reported in various experimental studies.[13–17]

Supplementary Table 1. Calculated BEEF-vdW adsorption and interaction energies, in eV, for the (111), (100), and (110) facets of both Pd and Pt metals. Adsorption energies of CO and O are given with respect to the gas phase molecules at low coverage, which we consider to be in the limit of zero monolayers. Adsorbate-adsorbate interactions are calculated as reported previously for binary gas-phase mixtures.[7]

Metal	Facet	$E_{ads,CO}$ (0ML)	$E_{ads,O}$ (0ML)	w_{CO}	w_O	$w_{CO,O}$
Pd	(111)	-1.734	-1.214	-0.168	-0.179	-0.133
	(100)	-1.666	-1.064	-0.149	-0.192	-0.150
	(110)	-1.727	-0.960	-0.159	-0.237	-0.105
Pt	(111)	-1.548	-1.090	-0.149	-0.158	-0.121
	(100)	-1.868	-1.166	-0.112	-0.101	-0.114
	(110)	-1.856	-1.197	-0.139	-0.207	-0.117

Supplementary References

1. Vendelbo, S. B. *et al.* Visualization of oscillatory behaviour of Pt nanoparticles catalysing CO oxidation. *Nat. Mater.* **13**, 884–890 (2014).
2. Kresse, G. & Furthmüller, J. Efficient iterative schemes for ab initio total-energy calculations using a plane-wave basis set. *Phys. Rev. B* **54**, 11169 (1996).
3. Kresse, G. Efficiency of ab-initio total energy calculations for metals and semiconductors using a plane-wave basis set. *Comput. Mater. Sci.* **6**, 15 (1996).
4. Kresse, G. & Hafner, J. Ab initio molecular dynamics for liquid metals. *Phys. Rev. B* **47**, 558–561 (1993).
5. Kresse, G. & Hafner, J. Ab initio molecular-dynamics simulation of the liquid-metalamorphous-semiconductor transition in germanium. *Phys. Rev. B* **49**, 14251–14269 (1994).
6. Blöchl, P. E. Projector augmented-wave method. *Phys. Rev. B* **50**, 17953 (1994).
7. Meng, J., Zhu, B. & Gao, Y. Shape Evolution of Metal Nanoparticles in Binary Gas Environment. *J. Phys. Chem. C* **122**, 6144–6150 (2018).
8. Zhu, B. *et al.* Reshaping of Metal Nanoparticles Under Reaction Conditions. *Angew. Chemie - Int. Ed.* **59**, 2171–2180 (2020).
9. Zhu, B., Meng, J. & Gao, Y. Equilibrium Shape of Metal Nanoparticles under Reactive Gas Conditions. *J. Phys. Chem. C* **121**, 5629–5634 (2017).
10. Zhang, X. *et al.* Unexpected refacetting of palladium nanoparticles under atmospheric N_2 conditions. *Chem. Commun.* **54**, 8587–8590 (2018).
11. Wellendorff, J. *et al.* Density functionals for surface science: Exchange-correlation model development with Bayesian error estimation. *Phys. Rev. B* **85**, 235149 (2012).
12. Dumesic, J. *et al.* The CO/Pt(111) Puzzle. *J. Phys. Chem. B* **105**, 4018–4025 (2002).
13. Conrad, H., Ertl, G., Koch, J. & Latta, E. E. Adsorption of CO on Pd single crystal surfaces. *Surf. Sci.* **43**, 462–480 (1974).
14. Behm, R. J., Christmann, K., Ertl, G. & Van Hove, M. A. Adsorption of CO on Pd(100). *J. Chem. Phys.* **73**, 2984–2995 (1980).
15. Yeo, Y. Y., Vattuone, L. & King, D. A. Energetics and kinetics of CO and NO adsorption on Pt{100}: Restructuring and lateral interactions. *J. Chem. Phys.* (1996).
16. Yeo, Y. Y., Vattuone, L. & King, D. A. Calorimetric heats for CO and oxygen adsorption and for the catalytic CO oxidation reaction on Pt{111}. *J. Chem. Phys.* **106**, 392–401 (1997).
17. Wartnaby, C. E., Stuck, A., Yeo, Y. Y. & King, D. A. Microcalorimetric heats of adsorption for CO, NO, and oxygen on Pt{110}. *J. Phys. Chem.* **100**, 12483–12488 (1996).
