# Supplementary material for: Structural changes in noble metal nanoparticles during CO oxidation and their impact on catalyst activity
Source: Nat Commun. 2020 May 1;11:2133. doi: 10.1038/s41467-020-16027-9 (PMC7195460; doi:10.1038/s41467-020-16027-9)
Supplement: Supplementary file 3 — Description of Additional Supplementary Files [file 41467_2020_16027_MOESM3_ESM.pdf]

Description of Additional Supplementary Files

File Name: Supplementary Movie 1
Description: Movie describing the structural changes in the Pd NP shown in Figures 1 and 2 during the heating ramp from 300 to 500 °C. The labeled temperature is the temperature set in the program. The image sequence has been drift-corrected, accelerated by 10×.

File Name: Supplementary Movie 2
Description: Movie describing the structural changes in the Pd NP shown in Figures 1 and 2 during the cooling ramp from 500 to 300 °C. The labeled temperature is the temperature set in the program. The image sequence has been drift-corrected and accelerated by 10×.

File Name: Supplementary Movie 3
Description: Movie describing the structural changes in several Pd NPs during a heating ramp from 300 to 500 °C. The labelled temperature is the temperature set in the program. The image sequence has been driftcorrected and accelerated by 10×.

File Name: Supplementary Movie 4
Description: : Movie describing the structural changes in the same Pd NPs captured in Supplementary Movie 3 during the cooling ramp from 500 to 300 °C. The labeled temperature is the temperature set in the program. The image sequence has been drift-corrected and accelerated by 10×.

File Name: Supplementary Movie 5
Description: Movie describing the structural changes in five Pd NPs during the heating ramp from 400 to 600 °C shown in Figure 3. The labeled temperature is the temperature set in the program. The image sequence has been drift-corrected and accelerated by 10×.

File Name: Supplementary Movie 6
Description: Movie describing the structural changes in the same Pd NPs captured in Supplementary Movie 5 during the cooling ramp from 600 to 400 °C as shown in Figure 3. The labeled temperature is the temperature set in the program. The image sequence has been drift-corrected and accelerated by 10×.
